# Supplementary figures and images for: Midazolam impacts acetyl—And butyrylcholinesterase genes: An epigenetic explanation for postoperative delirium?
Source: PLoS One. 2022 Jul 8;17(7):e0271119. doi: 10.1371/journal.pone.0271119 (PMC9269431; doi:10.1371/journal.pone.0271119)

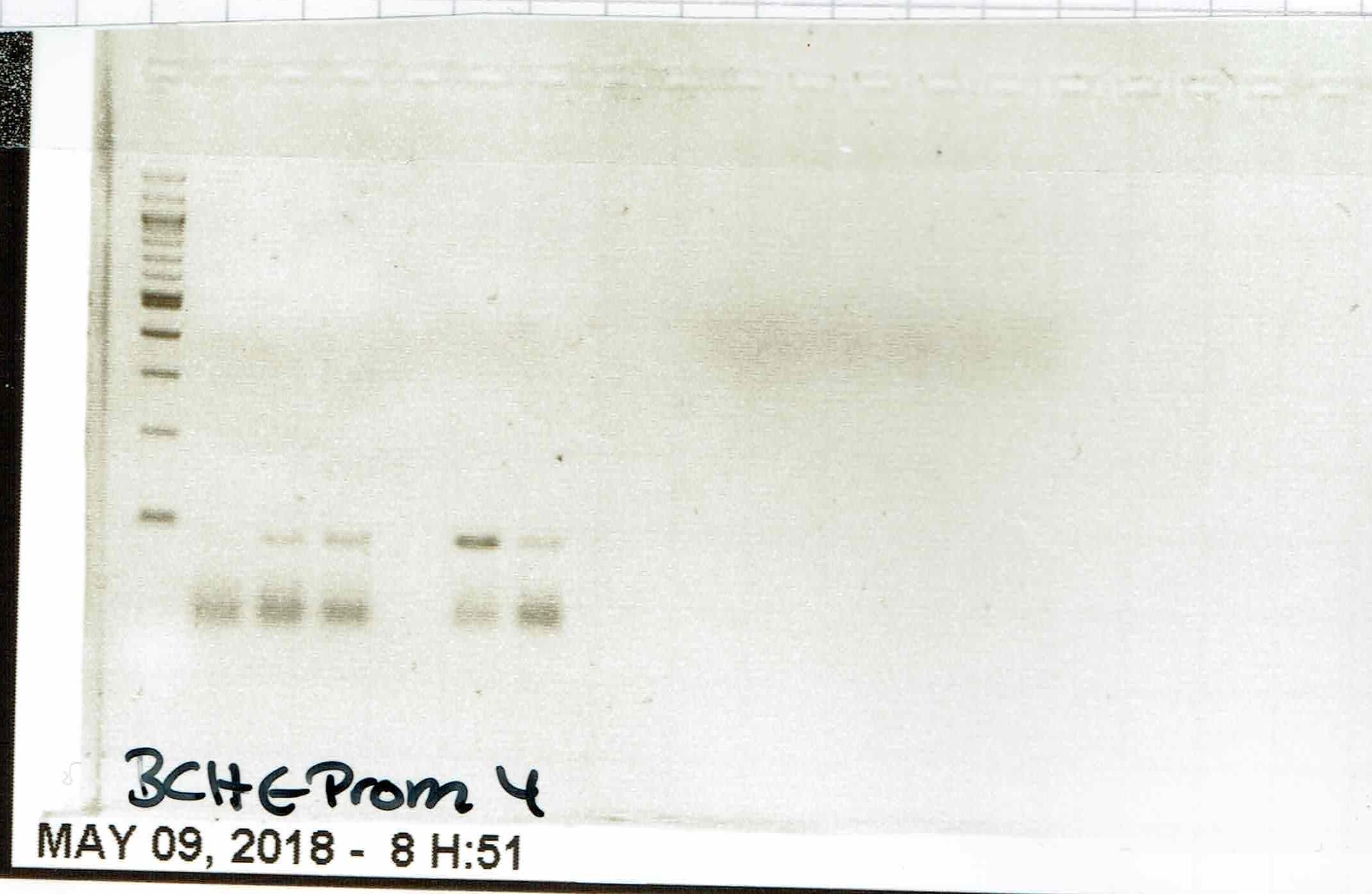

Supplement: S1 Raw images — (JPG) [file pone.0271119.s001.JPG]
