# Supplementary material for: Midazolam impacts acetyl—And butyrylcholinesterase genes: An epigenetic explanation for postoperative delirium?
Source: PLoS One. 2022 Jul 8;17(7):e0271119. doi: 10.1371/journal.pone.0271119 (PMC9269431; doi:10.1371/journal.pone.0271119)
Supplement: Attachment — Submitted filename: response to reviewers.docx [file pone.0271119.s002.docx]

Dr. rer. nat. Katharina Rump

Klinik für Anästhesiologie, Intensivmedizin und Schmerztherapie Universitätsklinikum Knappschaftskrankenhaus der Ruhr Universität Bochum

In der Schornau 23-25

D-44892 Bochum

Tel.: 0049234/3229242

Fax: 0049234/2993009

[Katharina.k.rump@rub.de](mailto:Katharina.k.rump@rub.de)

June, 14^th^ 2022

**Cover Letter for the review of the manuscript PONE-D-22-13550**

Dear Doctor Silman,

thank you very much for editing and evaluating our manuscript. Please find attached the revision of our original research article entitled “Midazolam impacts acetyl- and butyrylcholinesterase genes: An epigenetic explanation for postoperative delirium?” for consideration for publication in your journal.

We are pleased that you consider our manuscript to address an important topic, and we are happy to have addressed all comments raised by the reviewers, which has resulted in significant improvement of our manuscript. We have marked all changes in the manuscript as the “track-changes” option and explained point by point our responses to the reviewer’s suggestions in the following paragraph. In addition, we would like to state that: “The authors received no specific funding for this work”.

***Reviewer #1:*** Summary: Midazolam is given to patients before surgery. Midazolam is responsible for the delirium experienced by some patients in the days after surgery. Based on previous reports that cholinesterase activity levels are altered in patients who experience delirium, the present work hypothesized that midazolam affects the cholinesterase genes. Stable cell lines and peripheral blood mononuclear cells were assayed for the effect of midazolam on cholinesterase activity and mRNA levels, methylation of cholinesterase genes, demethylation enzyme activity, binding of the BCHE promoter to Histone and neuroinflammation. It was concluded that BCHE gene overexpression may aggravate postoperative delirium.

***In response:*** *We are pleased that you consider our manuscript to address an important topic, and we are happy to discuss the concerns you raised. Please find below a point-by-point reply addressing your remarks*.

1. Abstract. Please mention that your studies used human SH-SY5Y neuroblastoma cells, U343 glioblastoma cells, and human peripheral blood mononuclear cells.

***In response:*** *Thank you very much for this helpful comment. We now included into the abstract in lines 20 and 21:*

In an in-vitro model containing SH-SY5Y neuroblastoma cells, U343 glioblastoma cells, and human peripheral blood mononuclear cells, we found that midazolam altered the activity of acetylcholinesterase /buturylcholinesterase (AChE / BChE).

2. Abstract. Please list the methods used in your study.

***In response:*** *Thank you very much for this helpful comment. We now included into the abstract in lines 18 till 20:*

Consequently, we tested the hypothesis that midazolam indeed changes the expression and activity of cholinergic genes by acetylcholinesterase assay and qPCR. Furthermore, we investigated the occurrence of changes in the epigenetic landscape by methylation specific PCR, ChiP-Assay and histone ELISA.

3. You find that midazolam increased the level of BChE activity. However, references 16 and 18 report decreased BChE activity in the blood of patients treated with midazolam, on day 1 after surgery. Perhaps the abstract refers to increased BCHE mRNA rather than BChE activity.

***In response:*** *Thank you again for your valuable comment. Indeed, it is described in literature that BChE activity is decreased 48 hours after surgery, while AChE activity is not affected. In our study we could not differentiate between acetylcholesterase (AchE) or butyrylcholinesterase (BChE) activity as both enzymes can hydrolyze acetylcholine. However, we saw that BCHE mRNA expression slightly decreased after midazolam exposure, which was followed by an increase after 24 hours. Hence, this could depict a feedback mechanism for the initial decrease.*

4. Page 4 of 20, line 80. Typing error: 2.5% should be 0.25% Trypsin-EDTA

***In response:*** *We corrected this typing error.*

5. Page 4 of 20. Did you wash the cells with phosphate buffer saline before adding Trypsin-EDTA? This question is relevant to your assay of AChE activity because fetal bovine serum has substantial AChE activity. If you did not wash the cells, bovine AChE activity may be included in the AChE activity reported for cell lysates.

***In response:*** *Thank you very much for this comment. Yes, cell lysate preparation for AChE/BChE activity assay contained a wash step with PBS. We now included in line 111:*

The proteins were isolated as previously described (20) after washing the cells with PBS.

6. Please state the source of midazolam. Which midazolam salt did you use? What solvent was used to dissolve midazolam?

***In response:*** *We utilized midazolam hydrochloride injection solution from B. Braun, Melsungen. It is dissolved in 10 % HCl and sterile water. We now included in line 94:*

Briefly, cells were cultured in 6-well culture plates and incubated with 250 ng/ml, 1 µg/ml or 50 µg/ml midazolam (midazolam hydrochloride injection solution, B. Braun Melsungen) for 2, 4 and 24 h, 10 µg/ml and 50 µg/ml flumazenil or were left unstimulated (control).

7. Page 4 of 20, line 94. Please define the abbreviation BV-2.

***In response:*** *I am sorry for not providing you an abbreviation for this cell line. However, the accession number was now included in the test on line 229:* neural glial cells (BV-2, RRID:CVCL_0182 )

8. The word “stimulated” is used throughout the text when describing treatment with midazolam. What observation explains your choice of the word “stimulated”. Perhaps the cells proliferated more rapidly in the presence of midazolam?

***In response:*** *Thank you for this comment. I agree that “stimulate” is not neutral wording but implies a change in the cells biochemical processes. Indeed, we incubated the cells to examine putative stimulation. I changed my language and replaced the word “stimulated” by “incubated” or “treated” throughout the whole manuscript.*

9. Figure 1A. The y axis is labeled AChE/BChE activity, indicating AChE activity was divided by BChE activity. However, the assay measured total AChE and BChE activity because both enzymes hydrolyze the same substrate. Please change the y axis to Cholinesterase activity.

*Figure 1 has been changed.*

10. Figures 1B and 1C. The label on the y axis gives no clue that the assay measures mRNA level. Please modify the label on the y axis to AChE mRNA and BChE mRNA

*Figure 1 has been changed.*

11. The abbreviation ACTB is on the y axis of Figures 1B, 1C, , 2A, 2B, 4A, 4B, 4C, and 4D. Please replace ACTB with a word that is easy to understand. Perhaps the ACTB label is intended to mean that the reported mRNA quantity is relative to the mRNA for beta actin. This should be explained in the figure legend.

*The figures were changed and the legends of Figure 2 and 4 were modified.*

12. Page 8 of 20, line 178. It is suggested to write “the midazolam antagonist flumazenil reverses midazolam induced effects”

*This was changed.*

13. Figure 2 is missing the letters A and B for panels A and B.

*We changed this.*

14. Figure 2A please change the label on the y axis to AChE mRNA

15. Figure 2B please change the label on the y axis to BChE mRNA

*We changed this.*

16. Page 9 of 20. Line 186. Typing error Figure 1A should be Figure 2A.

*We changed this.*

17. Page 9 of 20, lines 194 to 196. Typing error. Figures 2A, 2B, 2C should be Figures 3A, 3B, 3C.

*We changed this.*

18. Figure 3 legend. The words lines and line should be lanes and lane.

*We changed this.*

19. Figure 3C. Please add numbers for DNA sizes. Of special interest are the sizes of the bands in lanes 1 to 7.

*We added the numbers for DNA size.*

20. Page 10 of 20, lines 226-227. Typing error. Figures 4A and 4B should be 5A and 5B.

*We changed this.*

21. Conclusion. Line 323. BChE activity is extremely low to almost undetectable in stable cell lines. Your assay measured total cholinesterase activity, which in effect is AChE activity. Please delete the word “activity” in line 323 because BChE activity was not measured.

*We changed this.*

*Again, thank you very much for carefully reading the manuscript. All points you raised were considered and the errors were corrected.*

***Reviewer #2:*** This interesting study pursued the epigenetic origin of delirium by focusing on methylation changes in the butyrylcholinesterase(BChE) promoter and seeking the corresponding methylase changes in blood cells from patients treated with Midazolam and in cultured human-originated neuroblastoma cells.The concept is a strength point of this study, but there are weakness points as well, as listed below.

***In response:*** *We are pleased that you consider our manuscript to address an important topic, and we are happy to discuss the concerns you raised. Please find below a point-by-point reply addressing your remarks.*

1. Why limit the search for methylation but avoid pursuit of microRNAs targeting the cholinesterase mRNA transcripts?

***In response:*** *Thank you very much for this outstanding comment. We agree that microRNA has a great potential to regulate cholinesterase mRNA expression. However, the search term “cholinesterase microRNA” yields 70 results in pub med. Hence, we conclude that it depicts a relatively good studied mechanism, while to our knowledge methylation of cholinergic genes was not studied at all. We therefore focused on DNA methylation analysis, but will include studying microRNA in our future studies regarding midazolam and cholinergic activity.*

2. What is the sex origin of the studied cells and what about the other sex? Pursuing a regulation process in half of humanity seems odd.

***In response:*** *Thank you very much for this interesting comment. We mainly studied SH-SY5Y cells, which are from a female origin. In addition, U-343MG cells were studied, which are from a male origin. To completely circumvent gender specific effects, we utilized PBMCs from healthy controls (5 female and 3 male probands), where no gender dependent effect could be seen. We now included gender distribution into the methods section in line 88 on page 4.*

3. Rather than presenting bar graphs, please shift the graphs into box plots displaying the variability.

***In response:*** *Thank you very much for this outstanding comment. We now changed all graphs.*

In addition, we have now added new data into figure 3 d, where in the previous version “data not shown” was stated.

The authors thank the reviewers for their critical evaluation of our study and for their constructive suggestions that helped significantly improve the correct understanding of our manuscript. We now hope to have addressed all raised issues sufficiently. We are looking forward to your decision and hopefully favorable response.

Sincerely yours

Katharina Rump (Corresponding author)
